# A Multicenter Survey Study of Lung Transplant Program Staffing

**DOI:** 10.1097/TP.0000000000004478

**Published:** 2022-12-21

**Authors:** Anil J. Trindade, Kaitlyn C. Chapin, Whitney D. Gannon, David B. Erasmus, Ciara M. Shaver

**Affiliations:** 1 Division of Allergy, Pulmonary, and Critical Care Medicine, Vanderbilt University Medical Center, Nashville, TN.; 2 Vanderbilt Transplant Center, Vanderbilt University Medical Center, Nashville, TN.

## Abstract

**Figure:**
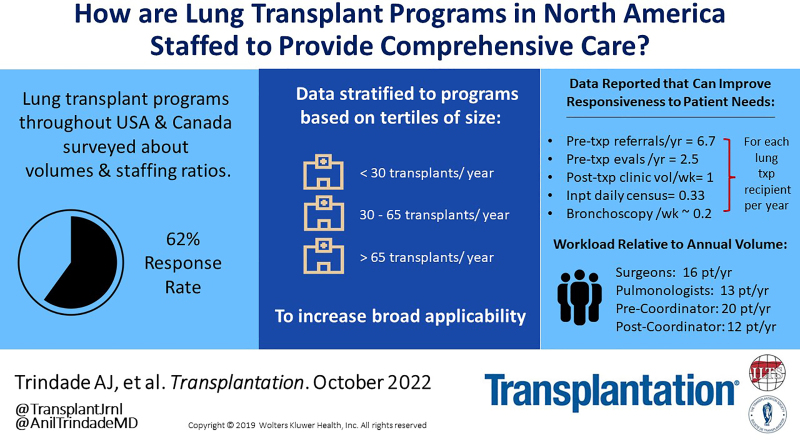

## INTRODUCTION

Approximately 2500 lung transplants are performed in the United States annually, yet there remains a significant shortage of suitable donor organs, resulting in lung transplant waitlist mortality of 15%.^1,2^ To meet demand, lung transplant programs are expanding in size and scope. Unfortunately, instruction on optimal program staffing structure is lacking. Furthermore, existing data are dichotomized into large (>40 transplants/y) or small (<40 transplant/y) programs, limiting program-specific interpretation for process improvement. Moreover, there are few details on procedure volumes, inpatient census, and planning for anticipated growth. Consequently, many lung transplant programs look to data in other solid organ fields for staffing guidance.^3–5^ However, such models may not be generalizable to the lung transplant community because lung allograft recipients have greater mortality, acute rejection, and comorbidities.^6,7^ To address this knowledge gap, we performed a prospective, multicenter, survey assessment of staffing in lung transplant programs across the United States and Canada. Program leadership was surveyed about clinical volumes, staffing adequacy, anticipated growth, and accommodations for coordinator workplace flexibility. These results provide a critical overview of staffing and identify key opportunities for improving logistics.

## MATERIALS AND METHODS

A 29-item survey (**Table S1**, **SDC**, http://links.lww.com/TP/C658) was administered via Research Electronic Data Capture to medical directors of active adult lung transplant programs in the United States and Canada identified through the Organ Procurement and Transplantation Network.^8^ Only 1 response was allowed per program. Weekly reminders were sent for 1 mo if there was no response.

Results were stratified into thirds by program size based on transplant procedures performed in 2021 with <30, 30–65, and >65 transplants/y defining small, medium, and large programs, respectively. Results were adjusted to 2021 transplant rates or total living cohort size, to facilitate comparisons between programs. Workload was calculated by dividing the metric of interest by number of full-time equivalents for the respective role; full-time equivalent equals scheduled hours divided by number of full-time workweek hours. Kruskal-Wallis testing was used to compare continuous variables across program sizes. Pairwise comparisons were performed using Mann-Whitney *U* and Chi-square testing for continuous and categorical variables, respectively. A *P* of <0.05 was considered statistically significant. Statistical analysis was performed using StataBE v17.0 (College Station, TX).

## RESULTS

### Patient Metrics by Program Size

**FIGURE 1.**
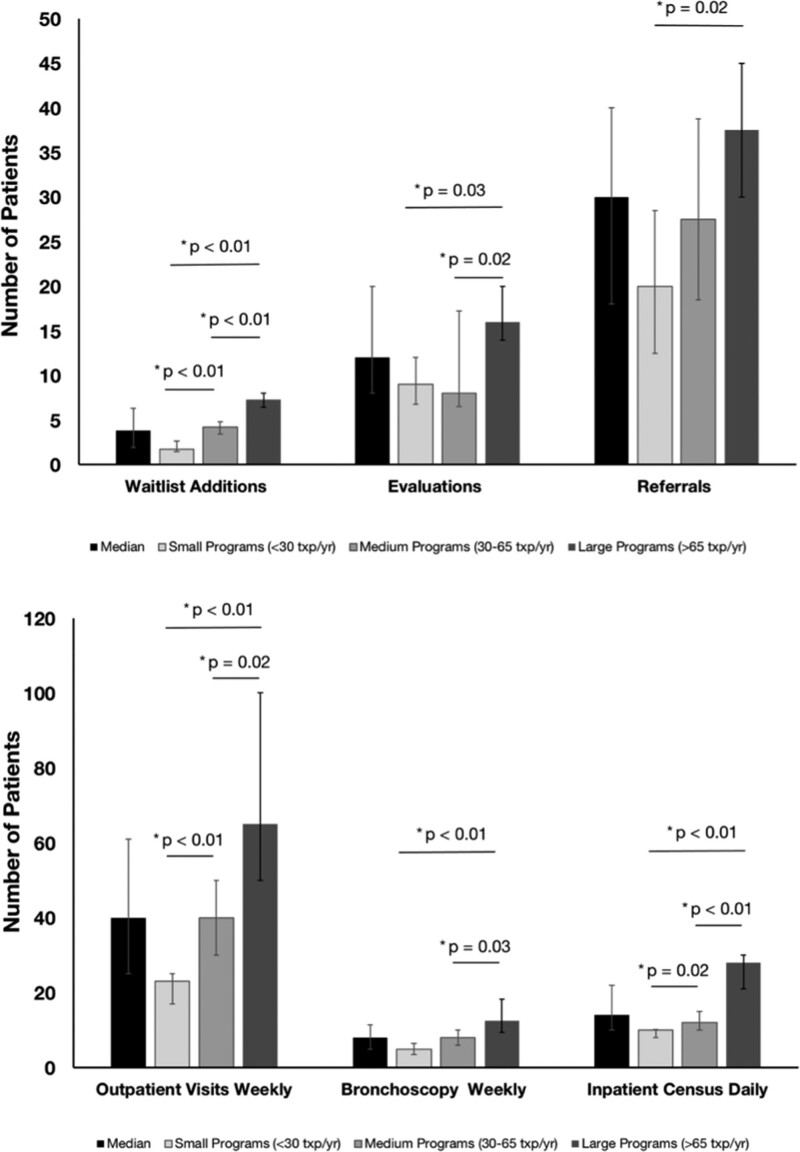
Transplant productivity by program size. A, Monthly waitlist additions, evaluations, and referrals stratified by program size. B, Outpatient clinic weekly volumes, bronchoscopy weekly volumes, and inpatient daily census, stratified by program size. For all panels, values are median with interquartile range. n = 11 small, 13 medium, and 15 large programs. Comparisons between program sizes were performed using Kruskal-Wallis testing or Mann-Whitney *U* testing. txp, transplant.

The survey was distributed to 63 active adult lung transplant programs, with 39 (62%) responding. Responses were received by 11 of 27 (41%), 13 of 19 (68%), and 15 of 17 (88%) of small, medium, and large programs, respectively. Medical directors (85%), other physicians (13%), or administrators (3%) provided responses. The median number of new transplants performed in 2021 was 46 (interquartile range 28–70). There was a significant difference in the number of transplants performed between small, medium, and large programs. **Table S2, SDC**, http://links.lww.com/TP/C658, summarizes the volume at each program, pretransplant metrics (referrals, evaluations, waitlist additions, new transplants), and posttransplant metrics (inpatient census, ambulatory visits, and bronchoscopy volume). Across all programs, 15% of referred patients were added to the transplant waitlist, whereas 40% of patients who completed evaluation were waitlisted (**Figure 1A**). There was no difference in progression from referral through evaluation and waitlisting by program size. Daily inpatient census was 33% of the annual transplant volume and was significantly higher for smaller programs (**Figure 1B**). The ratio of inpatient volume relative to the census of alive patients was 5% and did not vary by program size (*P* = 0.56). Ambulatory weekly volume was 103% of the annual transplant volume (14% of total living recipients) and did not vary by program size. Weekly bronchoscopy volume was 18% of annual transplant volume, with small programs performing more bronchoscopies than large programs (**Figure 1B**). Program metrics normalized to annual transplant volumes are in **Figures S1**, **S2, SDC**, http://links.lww.com/TP/C658.

### Staffing by Program Size

**FIGURE 2.**
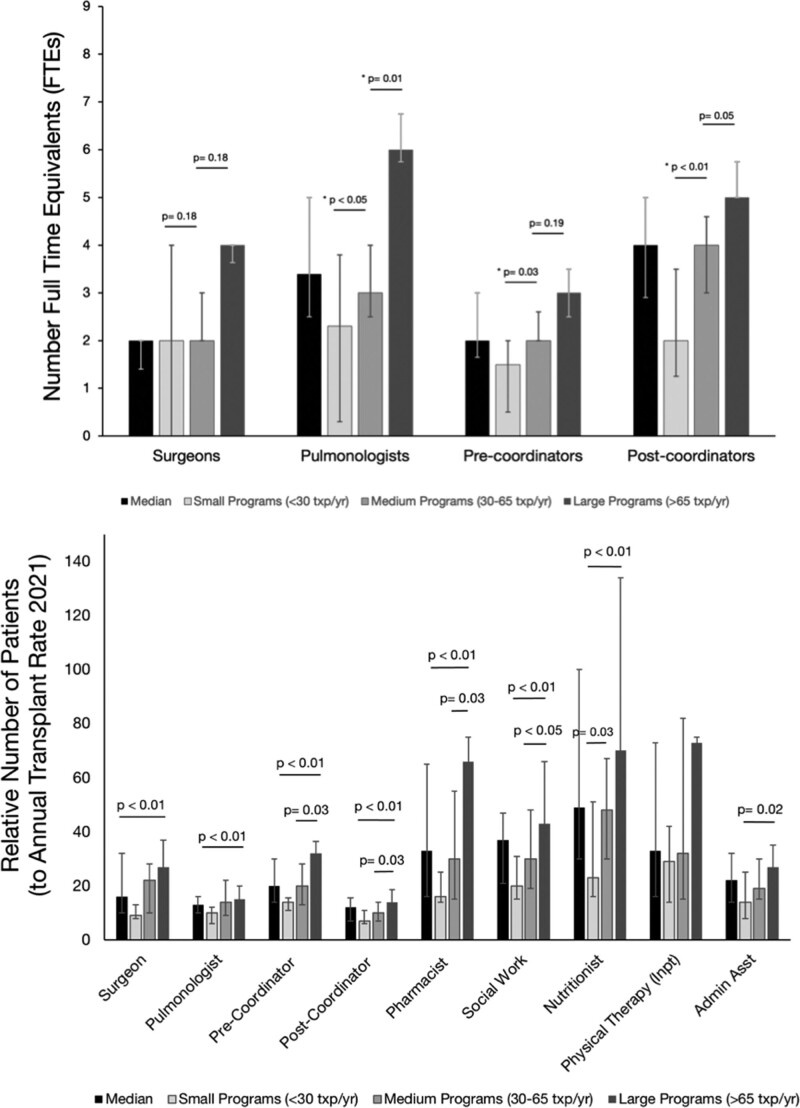
Staffing by transplant program size. A, Provider staffing stratified by program size. Number of full-time equivalents for select staffing roles is depicted using box and whisker plots showing median (horizontal bar), interquartile range (box), and SD (whiskers). B, Workload per full-time equivalent staff members, stratified by program size. Workload for staff members is shown relative to annual transplant volume per program. Only statistically significant results are shown. For all panels, values are median with interquartile range. n = 11 small, 13 medium, and 15 large programs. Comparisons between program sizes were performed using Kruskal-Wallis testing or Mann-Whitney *U* testing. FTE, full time equivalents; txp, transplant.

Professional staffing by each position is in **Table S3, SDC**, http://links.lww.com/TP/C658. There were significant differences in the number of surgeons, pulmonologists, inpatient advanced practice providers, coordinators, social workers, administrative assistants, and nutritionists by program size. There were no significant differences in the number of outpatient advanced practice providers, pharmacists, psychiatrists, or physical therapists by program size. There were significant differences in the number of pulmonologists and posttransplant coordinators between medium and large programs (**Figure 2A**). There were significant differences in the number of pulmonologists, pretransplant coordinators, and posttransplant coordinators between small and medium programs.

Relative workloads for each staff position are described in **Figure 2B** and **Table S4, SDC**, http://links.lww.com/TP/C658. Across all programs, there was 1 surgeon per 16 new lung transplants and 1 pulmonologist per 13 new transplants. Each posttransplant nurse coordinator provided care for a median of 77 patients, with 15% being within the first-year posttransplant. There were significant relative workload differences for pretransplant coordinators, posttransplant coordinators, pharmacists, and social workers between medium and large programs. The only role with a difference in workload between medium and small programs was nutritionists. When referenced to total cohort size, there was a relative workload difference between medium and large programs for postcoordinators and between small and large programs for pharmacists (**Figure S3, SDC**, http://links.lww.com/TP/C658).

### Perceived Staffing Adequacy by Program Size

Only 23% of programs perceived that current staffing resources were sufficient; most of these were large (**Table S5, SDC**, http://links.lww.com/TP/C658). The most understaffed positions reported were pulmonologist (36%), surgeon (23%), and posttransplant coordinator (10%). There were no differences in perceived sufficiency by program size. Despite perceptions of insufficient staffing, most programs (77%) planned to increase program volume within 5 y by a median of 37% (**Table S5, SDC**, http://links.lww.com/TP/C658).

### Remote Work Environment for Transplant Coordinators

The COVID-19 pandemic provided an impetus to improve workplace flexibility for transplant staff. Regardless of program size, most programs polled (59%) instituted remote capabilities for nurse coordinators. Most programs allowed for 40% of the work week to be spent working remotely (**Table S5, SDC**, http://links.lww.com/TP/C658). Although remote work options for nurse coordinators were numerically greater in large versus small programs (60% versus 20%), this difference was not statistically significant. Nursing and medical leadership had differing perspectives as to the ideal remote work schedule, with nursing suggesting that 2 d/wk (40%) was adequate, whereas medical leadership felt that 1 d/wk (20%) was sufficient (*P* = 0.04).

## DISCUSSION

In this article, we report current staffing practices across lung transplant programs in North America, using a prospective, multicenter survey study. We report significant differences in staffing between small, medium, and large transplant programs and identify significant differences in relative workload across different staff positions. These data will enable programs to adequately staff provider resources to meet lung transplant patient needs. Our study builds on limited United Network of Organ Sharing data by categorizing data into clusters based on program size, addressing inpatient and bronchoscopy volumes, and reflecting on anticipated program growth and coordinator workplace flexibility.

Our study provides key statistics to aid in logistical planning for lung transplant care delivery. We found that 15% of referred patients and 40% of patients completing transplant evaluations were waitlisted, findings that did not vary by program volume. Thus, to reach a set goal of transplants/year, transplant programs should plan referral visits for 7.5-times and transplant evaluations for 2.5-times as many patients as the goal number of transplants. These data can be used to assess needs for outreach and ensure that sufficient resources are dedicated to pretransplant workflow. We also report that inpatient daily census, weekly ambulatory clinic volumes, and weekly bronchoscopy needs are highly correlated with the annual transplant volume, which further enables advanced allocation of patient care resources.

This study provides important data on relative workloads of teams providing care for lung transplant patients. These data may help identify which additional resources will provide the best value during program expansion. Most programs reported plans to increase transplant volume by approximately one-third within 5 y, despite program leadership citing existing staffing insufficiencies. Although pulmonologist and surgeon roles were felt to be most needed, our data suggest greater variability in workload among coordinators, pharmacists, social workers, and nutritionists.

Finally, we highlight that most programs provide flexibility for nurse coordinators to work remotely at least 2 d (40%) of the week. Further assessment is necessary to determine the ideal balance between staff flexibility and patient outcomes.

Our study has multiple strengths. The 62% response rate shows that our data represent most North American transplant programs and exceeds the survey response rate of 30%–40% seen in similar studies.^3,4^ High participation was achieved because of the concise design, use of proactive weekly reminders to facilitate responses, and recognition among program leadership about the need for granular staffing data. Another strength is the breadth of representation of programs of different sizes. We also included workload assessments for a wide variety of clinical and nonclinical transplant team roles. Our study also has some limitations. Response rate of small programs was lower than large programs. In addition, we were unable to account for program-specific variation in staff role responsibilities; for example, pharmacists might have an outpatient presence in some programs, but only work with inpatient teams at others. It is also possible that staffing needs vary by patient demographics, geographic region, urban versus rural environment, patient travel distance, or involvement of medical housestaff. Notably, this work does not directly assess the influence of staffing ratios on patient outcomes, which would be an important natural extension in future studies.

In conclusion, our study provides a detailed description of staffing resources for lung transplant programs across North America, highlighting current staff workloads and providing critical data to provide logistical support for program optimization.

## Supplementary Material


