# Supplementary material for: A Multicenter Survey Study of Lung Transplant Program Staffing
Source: Transplantation. 2022 Dec 21;107(5):1013–6. doi: 10.1097/TP.0000000000004478 (PMC10125118; doi:10.1097/TP.0000000000004478)
Supplement: Supplementary file 1 [file tpa-107-1013-s001.pdf]

Supplemental Figures

Figure S1: Relative monthly referrals and evaluations needed to be performed for one waitlist addition. Values are referenced to the number of monthly waitlist additions. Values are median with interquartile range. n = 11 small, 13 medium, and 15 large programs. Comparisons between program sizes were performed using Kruskal-Wallis testing or Mann-Whitney U testing. txp, transplant; yr, year.

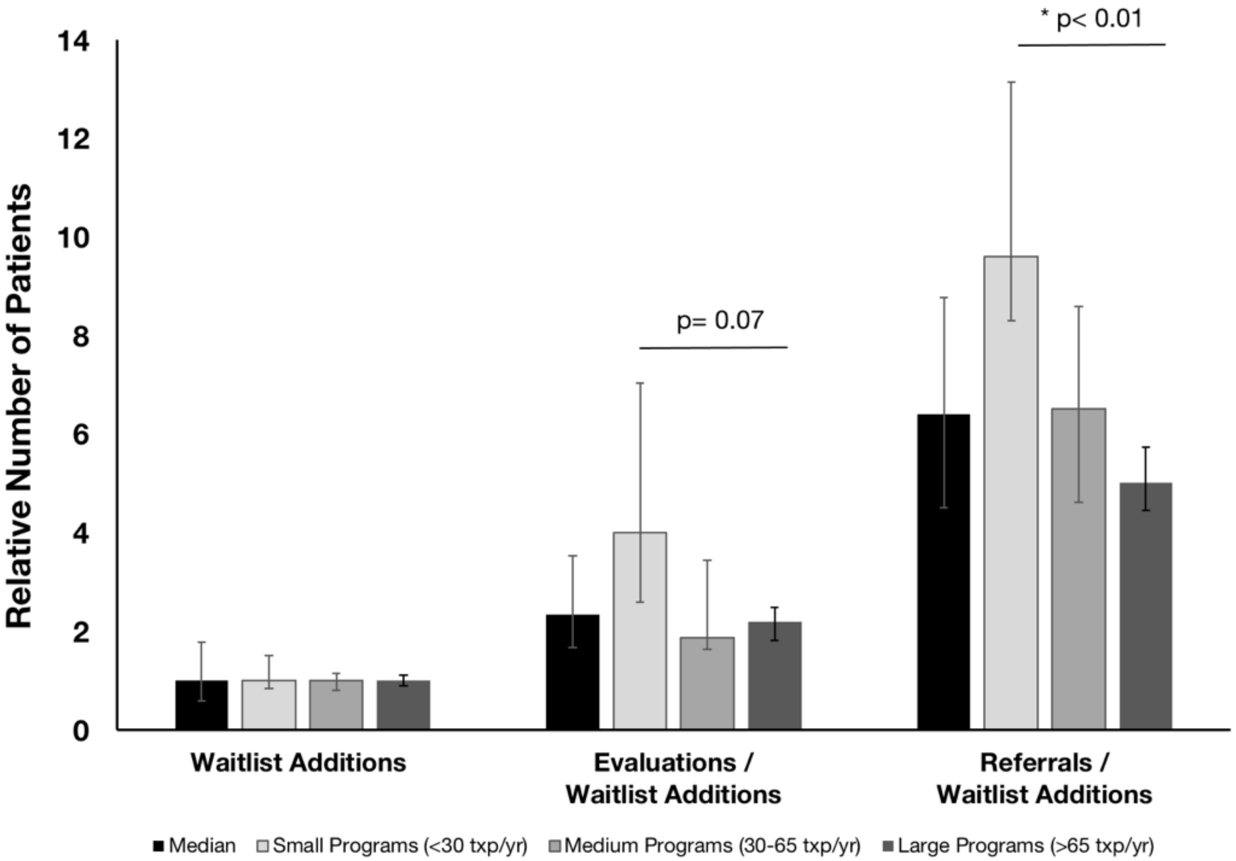

Figure S2: Inpatient and ambulatory patient volumes relative to annual transplant rate in 2021. Values are median with interquartile range. n = 11 small, 13 medium, and 15 large programs. Comparisons between program sizes were performed using Kruskal-Wallis testing or Mann-Whitney U testing. txp, transplant; yr, year.

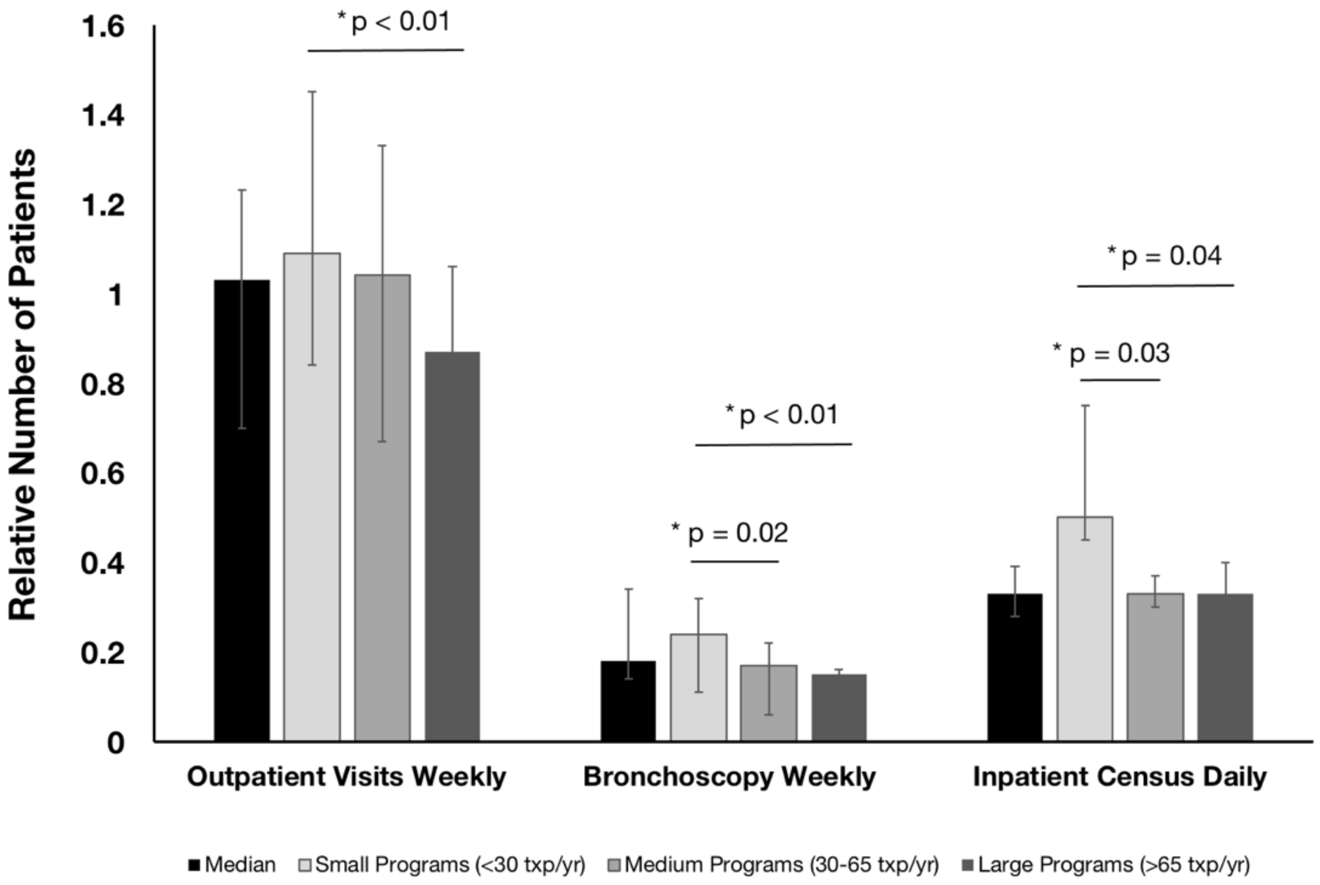

Figure S3: Workload per full-time equivalent relative to total cohort size, stratified by program size. Workload for staff members relative to total recipient cohort size at different sized transplant programs. n = 11 small, 13 medium, and 15 large programs. Comparisons between program sizes were performed using Kruskal-Wallis testing or Mann-Whitney U testing. Only statistically significant results are shown. txp, transplant; yr, year.

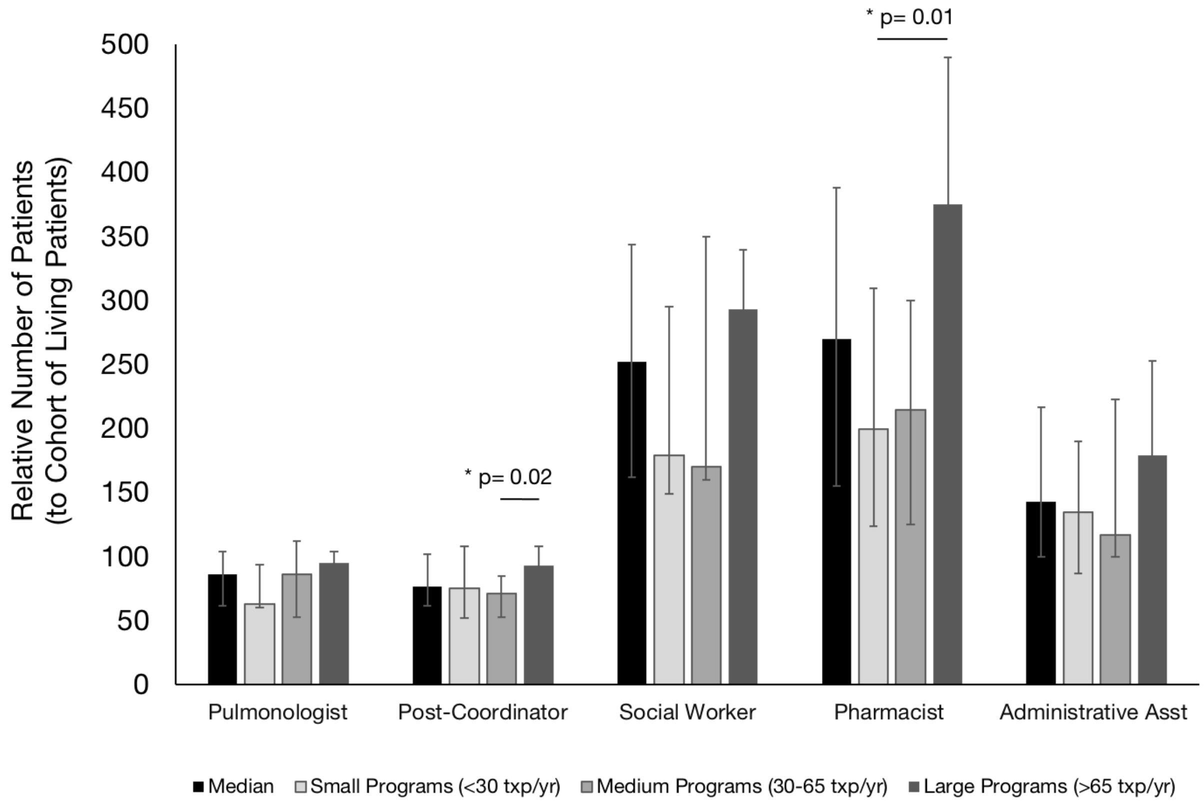

Supplemental Tables

Table S1: Lung transplant program survey questions.

1	Type of program / practice (academic, private/community hospital, mixed, other)
2	How many lung transplant procedures did your program perform in calendar year 2021?
3	How many lung transplant procedures did your program perform in calendar year 2020?
4	How many living transplant recipients is your program currently providing care for?
5	What model for follow-up care best describes your practice? [a) assume all aspects of care posttransplant, b) assume aspects of lung care posttransplant, c) provide lung care for first 1-2 years then defer to referring pulmonologist, d) other]
6	How many lung transplant procedures does your team wish to perform annually in the next 3-5 years?
7	How many referrals for lung transplantation does your program receive each month?
8	How many lung transplant evaluations does your program perform each month?
9	What is your average weekly clinic volume for posttransplant patients?
10	What is your average weekly clinic volume for new pretransplant patients?
11	What is your average daily inpatient lung transplant census?
12	How many lung transplant bronchoscopy procedures are performed each week?
13	How many full-time equivalent surgeons does your program utilize?
14	How many full-time equivalent pulmonologists does your program utilize?
15	How many full-time equivalent outpatient nurse practitioners / physician assistants does your program utilize?
16	How many full time equivalent inpatient nurse practitioners / physician assistants does your program utilize?
17	How many full-time equivalent pretransplant nurse coordinators does your program utilize?
18	How many full-time equivalent posttransplant nurse coordinators does your program utilize?
19	How many full-time equivalent administrative assistants (front desk staff) does your program utilize?
20	How many full-time equivalent social workers does your program utilize?
21	How many full-time equivalent pharmacists does your program utilize?
22	How many full-time equivalent psychiatry staff does your program utilize?
23	How many full-time equivalent nutritionists does your program utilize?
24	How many full-time equivalent physical therapists does your program utilize?
25	Do you feel that your current staffing model is sufficient?
26	What position on your team are you most in need of filling / increasing within the next 1-2 years (if any)?
27	Approximately what percentage of the work week are your nurse coordinators working from home?
28	What does your average nurse coordinator think is the ideal balance of working from home vs working in the office?
29	What do you think (ie, medical leadership) is the ideal ratio of working at home vs in

	the office for nurse coordinators?

Table S2: Patient volume by program size, median (interquartile range).

	**All programs (N = 39)**	**Small (N = 11)**	**Medium (N = 13)**	**Large (N = 15)**	***P* (All sizes)**	***P* (Between small to medium)**	***P* (Between medium to large)**
New transplants 2021	46 (28–70)	20 (15–25)	40 (30–48)	74 (70–91)	<0.01	<0.01	0.01
New transplants 2020	43 (29–67)	20 (15–24)	38 (35–48)	69 (64–108)	<0.01	<0.01	<0.01
Waitlist additions per month	4 (2–6)	2 (1–2)	4 (3–4)	7 (6–8)	<0.01	<0.01	<0.01
Evaluations per month	12 (8–20)	9 (7–12)	8 (7–17)	16 (14–20)	<0.01	0.33	0.02
Referrals per month	30 (18–40)	20 (13–29)	28 (19–39)	38 (30–45)	<0.01	0.09	0.42
Ratio listed / evaluated patients	0.40 (0.25–0.56)	0.25 (0.14–0.39)	0.54 (0.29–0.61)	0.43 (0.33–0.49)	0.22	0.12	0.47
Ratio listed / referred patients	0.15 (0.10–0.20)	0.10 (0.08–0.12)	0.16 (0.12–0.22)	0.19 (0.15–0.22)	0.06	0.10	0.39
Outpatient volume weekly	40 (25–61)	23 (17–25)	40 (30–50)	65 (50–100)	<0.01	0.23	0.42
Output volume (wk) / transplant rate in 2021	1.03 (0.70–1.23)	1.09 (0.84–1.45)	1.04 (0.67–1.33)	0.87 (0.61–1.06)	0.16	0.40	0.32
Outpatient volume (wk) / alive census	0.14 (0.11–0.17)	0.12 (0.10–0.15)	0.14 (0.11–0.19)	0.15 (0.13–0.17)	0.18	0.27	0.69
Inpatient census daily	14 (10–22)	10 (8–10)	12 (10–15)	28 (22–30)	<0.01	0.02	<0.01
Inpatient census / transplant rate in 2021	0.33 (0.29–0.49)	0.50 (0.37–0.58)	0.33 (0.22–0.38)	0.30 (0.28–0.33)	0.01	0.03	0.45
Inpatient census / total alive cohort	0.05 (0.04–0.08)	0.05 (0.05–0.09)	0.05 (0.04–0.06)	0.04 (0.04–0.08)	0.56	0.41	1.0
Bronchoscopy volume weekly	8 (5–12)	5 (4–7)	8 (6–10)	13 (10–18)	<0.01	0.07	0.03
Bronch volume (wk) / 2021 TXP volume	0.18 (0.13–0.24)	0.24 (0.19–0.49)	0.17 (0.14–0.21)	0.15 (0.10–0.22)	0.04	0.06	0.47
Total alive cohort	300 (187–430)	150 (113–200)	275 (204–335)	515 (400–698)	<0.01	<0.01	<0.01

TXP, transplant; wk, week.

Table S3: Lung transplant program staffing (full-time equivalents) based on program size.

	**All programs (N = 39)**	**Small (N = 11)**	**Medium (N = 13)**	**Large (N = 15)**	***P*** **(All sizes)**	***P*** **(Small vs medium)**	***P*** **(Medium vs large)**
Transplant surgeons	2.0 (2.0–3.3)	2.0 (1.6–2.0)	2.0 (2.0–3.0)	4.0 (2.0–6.0)	**0.01**	0.18	0.19
Pulmonologists	3.4 (2.5–5)	2.3 (2.0–3.0)	3.0 (2.5–4.0)	6.0 (4.0–7.5)	**<0.01**	**<0.05**	**0.01**
Outpatient advanced practice providers	1.0 (0.5–1.4)	1.0 (0.5–1.0)	1.0 (1.0–2.0)	1.0 (0–1.5)	0.48	0.16	0.49
Inpatient advanced practice providers	2.0 (1.0–4.0)	1.0 (0.5–2.0)	3.0 (1.5–4.0)	4.0 (2.0–6.0)	**<0.05**	0.05	0.28
Pretransplant nurse coordinators	2.0 (1.7–3.0)	1.5 (1.0–2.0)	2.0 (2.0–2.6)	3.0 (2.0–3.5)	**<0.01**	**0.03**	0.19
Posttransplant nurse coordinators	4.0 (2.9–5.0)	2.0 (2.0–2.8)	4.0 (3.0–4.6)	5.0 (4.3–6.5)	**<0.01**	**<0.01**	0.05
Administrative assistants	2.0 (1.0–3.0)	1.0 (1.0–2.0)	2.0 (1.8–3.0)	3.0 (3.0–4.0)	**0.02**	0.11	0.09
Social workers	1.0 (1.0–2.0)	1.0 (0.7–1.0)	1.0 (1.0–2.0)	1.5 (1.0–2.0)	**<0.01**	0.06	0.26
Pharmacists	1.0 (1.0–2.0)	1.0 (1.0–1.0)	1.0 (1.0–2.0)	2.0 (1.0–2.0)	0.16	0.27	0.65
Psychiatry	0.55 (0.0–1.0)	0.2 (0.0–0.7)	0.6 (0.25–1.0)	1.0 (0.0–1.0)	0.11	0.09	0.68
Nutrition	1.0 (0.5–1.0)	1.0 (0.3–1.0)	1.0 (0.5–1.0)	1.0 (0.8–1.8)	0.30	0.68	0.13
Physical therapy (inpatient)	1.0 (0.2–2)	0.75 (0.5–1.0)	0.6 (0–2.0)	1.5 (1.0–2.5)	0.15	0.84	0.21

Data expressed as median values (interquartile range).

Table S4: Calculated relative staff workload.

	Surgeons	Pulmonologists	Pretransplant coordinators	Posttransplant coordinators	Pharmacists	Social work	Nutrition	Physical therapy (inpatient)	Administrative assistant
**Relative to lung transplant procedures performed in 2021**									
All (N = 39)	16 (10-32)	13 (10-16)	20 (14-30)	12 (7-15)	33 (16-65)	37 (21-47)	49 (30-90)	33 (16-74)	22 (14-32
Large (>65 txp/yr) (N = 15)	27 (15-37)	15 (12-20)	32 (23-37)	14 (12-19)	66 (34-75)	43 (37-70)	70 (52-134)	73 (28-76)	27 (22-36)
Medium (30-65 txp/yr) (N = 13)	22 (10-28)	14 (9-22)	20 (13-28)	10 (7-14)	30 (15-55)	30 (19-48)	48 (30-67)	32 (15-82)	19 (15-30)
Small (<30 txp/yr) (N = 11)	9 (8-13)	10 (6-12)	14 (11-15)	7 (6-11)	16 (14-24)	20 (15-31)	23 (16-51)	29 (14-42)	14 (8-25)
**Relative to total census of alive posttransplant patients**									
All (N = 39)		86 (62-104)		77 (62-102)	270 (155-388)	252 (162-344)			143 (100-217)
Large (>65 txp/yr) (N = 15)		95 (76-104)		93 (76-108)	375 (278-490)	292 (250-341)			179 (135-253)
Medium (30-65 txp/yr) (N = 13)		86 (53-112)		71 (53-85)	215 (125-300)	170 (160-350)			117 (100-223)
Small (<30 txp/yr) (N = 11)		63 (60-94)		75 (52-108)	200 (124-310)	179 (149-295)			135 (87-190)

Data expressed as median values (interquartile range).
txp, transplant; yr, year.

Table S5: Projected growth, perceived transplant staffing sufficiency, and coordinator flexibility.

	All programs (N = 39)	Small (N = 11)	Medium (N = 13)	Large (N = 15)	*P*
New transplant volume 2021	46 (28–70)	20 (15–25)	40 (30–48)	74 (70–91)	<0.01
Goal annual volume in 3-5 years	60 (40–88)	33 (26–40)	60 (50–60)	100 (80–100)	<0.01
Ideal projected growth	+37% (20%–57%)	+79% (25%–188%)	+45% (33%–58%)	+23% (7%–38%)	<0.01
Programs with sufficient staffing	9 (23%)	1 (9%)	3 (23%)	5 (33%)	0.35
**Most needed position to grow program**					
Transplant surgeon	9 (23%)	2 (18%)	6 (46%)	1 (7%)	0.04
Pulmonologist	14 (36%)	3 (27%)	4 (31%)	6 (40%)	0.77
Outpatient advanced practice provider	3 (8%)	0	1 (8%)	2 (13%)	0.85
Inpatient advanced practice provider	2 (5%)	1 (9%)	0	1 (7%)	0.92
Pretransplant nurse coordinator	3 (8%)	0	1 (8%)	2 (13%)	0.85
Posttransplant nurse coordinator	4 (10%)	2 (18%)	0	2 (13%)	0.74
Pharmacist	3 (8%)	1 (9%)	1 (8%)	1 (7%)	0.95
Physical therapist	1 (3%)	1 (9%)	0	0	0.88
**Nurse coordinator work-from-home**					
Programs with flexible coordinator schedules	23 (59%)	7 (64%)	7 (54%)	9 (60%)	0.88
Current % of week working remotely	40% (20%–60%)	20% (20%–40%)	40% (30%–50%)	60% (40%–80%)	0.82
Ideal remote work week (nurse coordinator perspective)	40% (20%–40%)	20% (20%–40%)	20% (0%–40%)	40% (20%–80%)	0.30
Ideal remote work week (medical leadership perspective)	20% (0%–40%)	20% (0%–20%)	20% (20%–40%)	20% (10%–40%)	0.22

Continuous variables expressed as median (interquartile range). Categorical variables are expressed as number (frequency).
