# Supplementary figures and images for: A Multicenter Survey Study of Lung Transplant Program Staffing
Source: Transplantation. 2022 Dec 21;107(5):1013–6. doi: 10.1097/TP.0000000000004478 (PMC10125118; doi:10.1097/TP.0000000000004478)

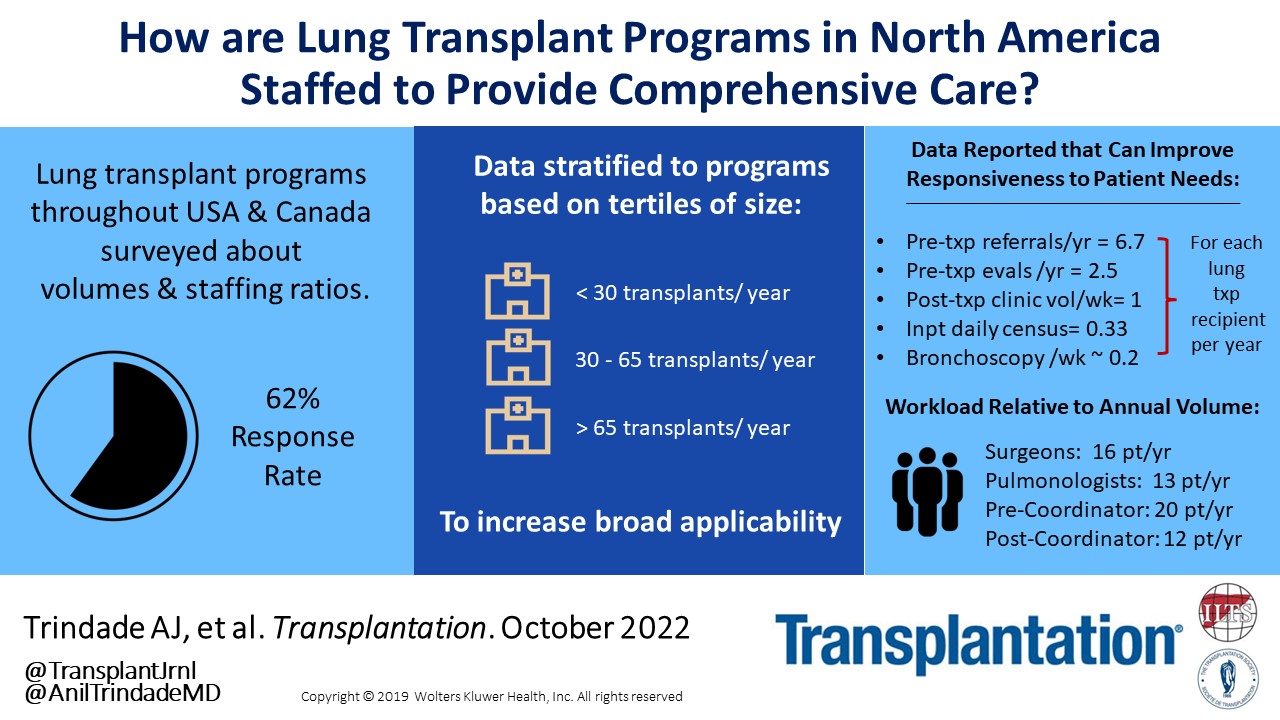

Supplement: Supplementary file 2 [file tpa-107-1013-s002.jpg]
